# Female cancer survivors’ return-to-work: Japanese situation

**DOI:** 10.20407/fmj.2022-017

**Published:** 2022-05-25

**Authors:** Atsuhiko Ota, Masaaki Matsunaga

**Affiliations:** Department of Public Health, Fujita Health University, School of Medicine, Toyoake, Aichi, Japan

Seko et al. reported that female outpatients with cancer in Japan were more likely to have the intention to work and seek regular employment than the general female population.^1^ Here, we introduce the current Japanese situation regarding return-to-work among cancer survivors.

Among those aged 20–54 years, the incidence of cancer is higher in females than in males.^2^ Along with breast cancer, uterine, ovarian, and thyroid cancers are common, with particularly high incidence rates among those aged under 35 years (Table 1). Given the high 5-year relative survival rates for breast, uterine, and thyroid cancers,^3^ it is a natural consequence that working-age female cancer survivors have the intention to work and seek regular employment. Existing evidence reported return-to-work rates ranging from 45.0% to 89.7% for breast cancer and from 42.9% to 95.2% for female patients with genital cancer in Japan.^4^

Females are vulnerable in the labor market in Japan and face more difficulty finding regular employment than males. The employment rate of females aged 15–64 years is lower than that for males of the corresponding age (70.6% vs. 83.8% in 2020).^5^ The proportion of non-regular employment is also much higher in females than males (54.4% vs. 22.2% in 2020).^5^ The Japanese Government asked employers to introduce social arrangements for working conditions to allow cancer survivors to balance their work and treatment, such as a policy statement by the employer, sick-leave programs, and working styles.^2,6^ However, some enterprises, especially small and medium-sized businesses, have not yet completed such arrangements.^7^ This could impact return-to-work among cancer survivors.

After cancer survivors return to work as intended, improvement in their physical and mental health may not be automatically ensured. A Japanese study found that working cancer survivors reported deteriorated self-rated health and limitations in physical functional capacity more frequently than workers without cancer.^8^ It is a matter of debate whether returning to work improves mental health among cancer survivors.^9^ Continuous health support is necessary, including after a cancer survivor returns to work.

## Figures and Tables

**Table1:** Cancer incidence for females in Japan by age in 2017 and 5-year relative survival rates

	All cancers n	Breast n (%)	Uterus n (%)	Ovary n (%)	Thyroid n (%)
Cancer incidence (Proportion to all)					
Age, years 20–24	843	52 (6.2)	46 (5.5)	168 (19.9)	215 (25.5)
25–29	1728	236 (13.7)	241 (13.9)	266 (15.4)	380 (22.0)
30–34	3850	951 (24.7)	878 (22.8)	343 (8.9)	564 (14.6)
35–39	7586	2696 (35.5)	1502 (19.8)	510 (6.7)	859 (11.3)
40–44	15,172	6986 (46.0)	2211 (14.6)	1000 (6.6)	1125 (7.4)
45–49	22,260	10,852 (48.8)	3049 (13.7)	1423 (6.4)	1134 (5.1)
50–54	23,186	9020 (38.9)	3421 (14.8)	1451 (6.3)	1062 (4.6)
55–59	26,072	8417 (32.3)	3281 (12.6)	1363 (5.2)	1137 (4.4)
60–64	33,273	9593 (28.8)	2856 (8.6)	1327 (4.0)	1202 (3.6)

5-year relative survival rate	66.9%	92.3%	78.7%	60.0%	95.8%

Data retrieved from references nos. 2 and 3. The numbers of all cancers included cancers other than breast, uterine, ovarian, and thyroid cancers. The 5-year relative survival rates were calculated using those who were diagnosed with cancer from 2009 to 2011.
